# Retail customers’ satisfaction with banks in Greece: A multicriteria analysis of a dataset

**DOI:** 10.1016/j.dib.2021.106915

**Published:** 2021-02-26

**Authors:** Dimitrios Drosos, Michalis Skordoulis, Nikolaos Tsotsolas, Grigorios L. Kyriakopoulos, Eleni C. Gkika, Faidon Komisopoulos

**Affiliations:** a University of West Attica, 250 Thivon and Petrou Ralli, GR12244 Egaleo, Greece; b Democritus University of Thrace, 193 Pantazidou, GR68200 Orestiada, Greece; c National Technical University of Athens, 15780 Athens, Greece

**Keywords:** Banking sector, Retail banking, Customer satisfaction, Multicriteria satisfaction analysis

## Abstract

The aim of this article is to present data concerning customers’ satisfaction with the 4 systemic banks operating in Greece. Today, the market share of these banks is over 95%, as the economic crisis of 2010 has led to structural changes in the banking industry of the country. At the same time, the conditions created by the COVID-19 pandemic will potentially lead to additional changes such as a more intensive use of alternative networks. To collect the research data, 5,018 questionnaires have been responded by retail customers of the 4 systemic banks operating in Greece during the period between June 2017 and June 2019. The criteria used to determine customers’ satisfaction with the 4 systemic banks of banks concern products and services, branch network, staff and customer service. Τhe collected data have been analyzed using the Muticriteria Satisfaction Analysis (MUSA) method. The results of the MUSA method provide several evidence that can be taken into account during the strategic development process of any organization including banks.

## Specifications Table

**Table:** 

Subject	Management Science and Operations Research
Specific subject area	Customer satisfaction
Type of data	Table, Figure
How data were acquired	The data were acquired using an electronic questionnaire which was delivered to banks’ retail customers.
Data format	Raw data Analysed data
Parameters for data collection	The data were collected from all around Greece, as the questionnaire was delivered electronically.
Description of data collection	The data were collected in four consecutive semesters (June 2017 - June 2019); 5018 valid questionnaires were collected, initially answered by 1255 Greek banks’ retail customers. A response rate of 8% is recorded. The questionnaire is provided as a supplementary file.
Data source location	Institution: University of West Attica Department: Department of Business Administration Address: 250 Thivon and Petrou Ralli, GR12244, Egaleo Country: Greece
Data accessibility	https://data.mendeley.com/datasets/8ghbmydv77/1

## Value of the Data

• Customer satisfaction is one of the most important strategic components any firm can use in order to succeed. Thus, the research data can be used by the banks’ decision makers to measure and understand their customers’ satisfaction. Furthermore, the research data can be used in order to analyze how customers’ satisfaction is correlated with other factors. A strengths-weaknesses-opportunities-threats (SWOT) analysis based on customer satisfaction will become feasible as well. Thus, ways to improve banks’ customer satisfaction will be pointed out.
• The data concern a period of intense changes in the banking industry of Greece. This period ended right before a potential new economic crisis due to the COVID-19 pandemic, meaning that a new period of intense changes and instability will begin. In such a period, the banks can rely on their customers’ satisfaction and loyalty to survive and succeed in the new environment. In this sense, the research data provide very useful information to banks’ decision makers.
• Both the banks’ decision makers and researches can benefit from the research data and their analysis.
• The research data can be compared with the cases of other countries in order to find possible similarities or differences.
• The questionnaire including satisfaction criteria and subcriteria can be used as an annual barometer of customer satisfaction in the Greek banking sector.

## 1 Data Description

Customer satisfaction is found to be correlated with firms’ competitive advantage, market share and financial performance [1,2]; thus, it is considered as one of the most important strategic components for any firm attempting to succeed and survive in today's fierce competitive environment. At the same time, a growing number of firms choose customer satisfaction as one of their main performance indicators [3]. As far as it is concerned, customer satisfaction is in the spotlight of both the researchers and the decision makers. Customer satisfaction is of high importance in the banking sector as well. In this context, several studies have been carried out, while different customer satisfaction scales have been used. BSQ (Bank Service Quality) scale was developed to measure the perceived service quality in retail banking based on 31 items of service quality categorized into 6 dimensions; these dimensions concern effectiveness and assurance, access, price, tangibles, service portfolio and, reliability [4]. SERVQUAL (Service Quality) scale, which determines reliability, empathy, responsiveness, assurance and tangibles as the service quality dimensions is extensively used to measure customers’ satisfaction with banks as well [5]. In the existing literature, similar dimensions have been used to determine customers’ satisfaction with the Greek banks [6].

Over the past decade, a major restructuring took place in the Greek banking industry due to the financial crisis that erupted in 2010 [7]. During this financial crisis, all the Greek banks faced difficulties concerning their capital adequacy and their toxic securities. It is noteworthy that while in 2012, there were 18 commercial banks operating in Greece, today there are only 4 systemic banks (National Bank of Greece, Piraeus Bank, Eurobank and Alpha Bank) which cumulatively account for more than 95% of the market, have almost the same market shares, and offer almost the same products and services [7,8]. Today, the banking industry in Greece is considered as one of most important sectors of the economy, since it significantly contributes to its reforming and supports its recovery [7,^9^,10]. However, due to the COVID-19 pandemic, further changes are possible in the industry, as temporary measures, such as the elimination of many transactions in the banks’ branches, would potentially become permanent, based on the cases of other countries [11].

In this research, customer satisfaction criteria and subcriteria are selected based on an extensive review of the relevant literature [4,^5^,^6^,^12^,^13^,^14^,15]. The main criteria selected to determine customer satisfaction concern products and services, branch network, staff and customer service. For each one of these criteria, a number of satisfaction subcriteria is selected to be measured as required by the MUSA method. The satisfaction criteria and subcriteria are provided in Table 1.

**Table 1:** Greek banks’ customer satisfaction criteria and subcriteria.

Satisfaction criteria (dimensions)	Satisfaction subcriteria
Products and services	• Products and services’ variety • Provided information • Problem solving efficiency • Problem solving time • Products and services’ quality • Procurements of products and services • Returns of products and services • Specialized services availability
Branch network	• Ease of access • Branch network area coverage • Branches’ atmosphere • Branches’ cleanliness • Branches’ layout • Staff adequacy • Waiting time • Service time • Cashiers’ number • ATMs’ and APSs’ number
Staff	• Staff kindness and willingness • Staff knowledge • Staff confidentiality • Staff skills • Staff image • Staff professionalism • Staff reliability
Customer service	• Complaints address immediacy • Customer service processes • Customer service immediacy • Information provision immediacy • Service provision without mistakes • Promises keeping • Search for the best possible solutions

Cronbach's alpha is used to estimate customer satisfaction dimensions reliability. Based on Table 2, we see that all the values are higher than 0.7 which is the lowest acceptable value; thus, very high internal consistency is indicated.

**Table 2:** Customer satisfaction dimensions reliability.

Satisfaction criteria (dimensions)	Cronbach's alpha
Products and services	0.855
Branch network	0.909
Staff	0.966
Customer service	0.941

**Fig. 1:**
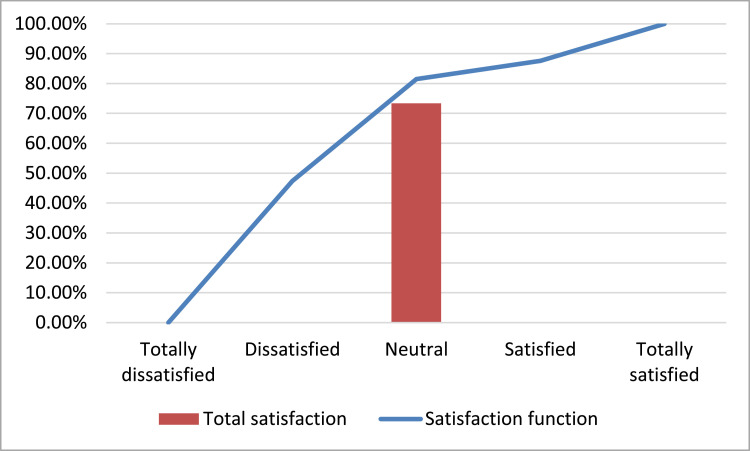
Satisfaction function and customers’ total satisfaction index.

The collected data show that Greek banks’ customers are demanding, as shown by the increasing trend of the MUSA method's satisfaction function [16], in Fig. 1. At the same time, customers’ total satisfaction index is equal to 73.35% during the examined period.

**Fig. 2:**
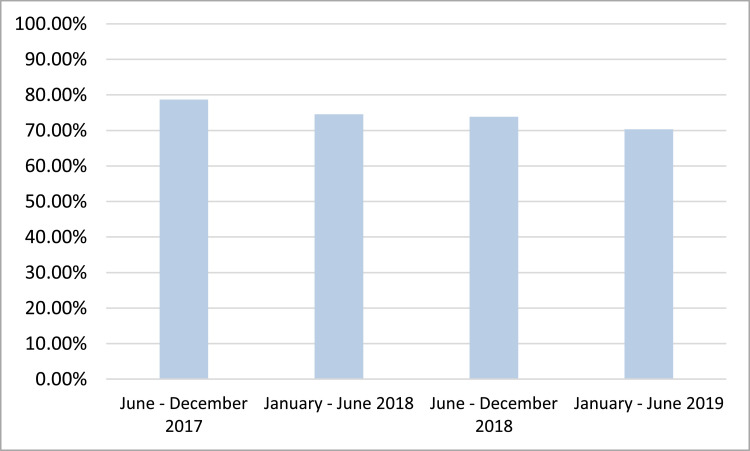
Level of customer satisfaction.

Based on Fig. 2, we see that during the examined period, the highest value of customer satisfaction is recorded between June and December 2017 (78.68%), while the lowest one is observed between January and June 2019 (70.35%). Thus, a negative trend is recorded. This trend can be adequately explained by the restructuring of the banking industry which has led to a reduction of both the banks’ staff by 23% in 2019 compared to 2015, and the banks’ branches by 28% during the same period [8,^17^,18]. Thus, this negative trend in customer satisfaction has been attributed to the fact that the competitive and localized advantage of in-person, both business-to-business (B2B) and business-to-consumer (B2C) modes of servicing, they have been either weakened or overly discontinued.

**Fig. 3:**
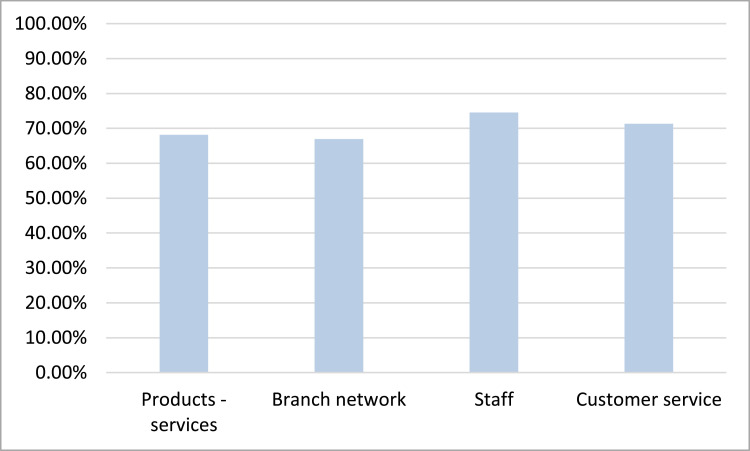
Satisfaction criteria performance.

As far as the satisfaction criteria are concerned, based on Fig. 3, we see that the criterion with the highest performance is this of staff (74.53%) while the criterion with the lowest performance is this of branch network (66,91%).

**Fig. 4:**
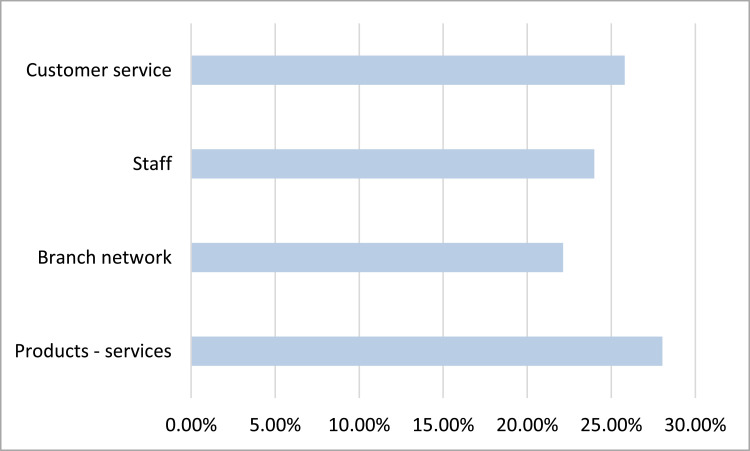
Satisfaction criteria importance.

Furthermore, based on Fig. 4, we that the most important criterion for customer satisfaction is this of the provided products and services (28.05%), while the least important one is the one concerning the branch network (22.13%).

**Fig. 5:**
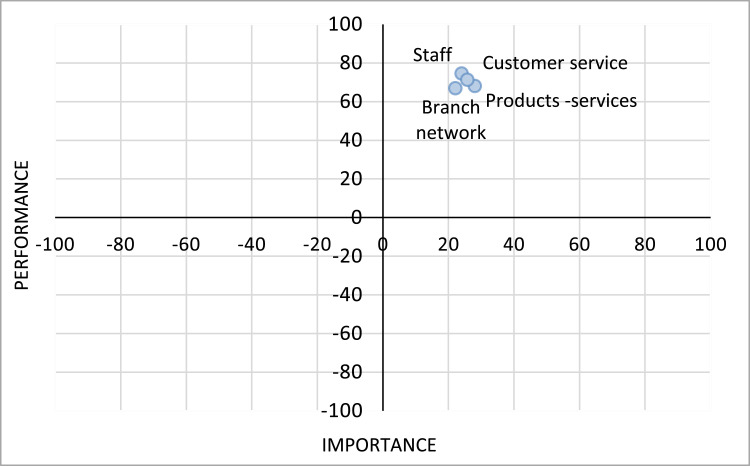
Satisfaction criteria action diagram.

The MUSA method results can be used for an organization's strategic development [16]. Such a result is the action diagram of Fig. 5. Based on this diagram we see that all the satisfaction criteria are placed in the area of high performance and importance (leverage opportunity area).

## 2 Experimental Design, Materials and Methods

The research data were collected in four consecutive semesters, between June 2017 and June 2019. In order to calculate a representative sample size, we referred to both the sample size calculation theory and the existing surveys measuring Greek banks customers’ satisfaction. Based on the sample size calculation theory [19], a sample size of 384 questionnaires is needed in the case of 5% sampling error, taking into account that the total population of Greek banks’ customers is about 7.5 million people. Concerning the relevant surveys, the sample size range is between 108 [14] and 1260 [15] questionnaires. Based on the above analysis we estimated a sample size of 1200 questionnaires. To obtain the needed sample we randomly approached 15,000 banks’ retail customers by delivering them an electronic questionnaire. The questionnaire was developed based on a 5 point Likert scale as required by the MUSA method. In the questionnaire's scale, 0 denotes totally dissatisfied, while 4 denotes totally satisfied. Random sampling was used as the same technique is used in all the relevant studies. This sampling method is used due to its ease of use and accuracy of representation in extracting samples from large populations. 1255 questionnaires were finally responded. Thus, a response rate of about 8% is recorded. Those who agreed to participate in the survey and returned the questionnaire, consented that they would respond to the same questionnaire for another 3 consecutive semesters. In this way, 5018 valid questionnaires were finally collected.

The MUSA method is used to measure customers’ satisfaction. This method uses data concerning customers’ satisfaction with a set of variables, based on the multicriteria nature of the quality of a product or a service. These variables are the satisfaction criteria and subcriteria. The MUSA method uses goal programming techniques and follows the general principles of qualitative regression analysis under constraints in order to assess global and partial satisfaction functions Υ* and X_i_ respectively, given customers’ ordinal judgments Y and X_i_. The additive utility model which is MUSA's core principal, is represented by the following equation of ordinal regression [16,20]:

(1) $${\tilde{Y}}^{*}=\sum_{i=1}^{n}b_{i}x_{i}^{*}-\sigma^{+}+\sigma^{-}$$

where:

• ${\tilde{Y}}^{*}$: is the estimation of the global value function,
• n: represents the number of criteria,
• b_i_: is a positive weight of the *i*th criterion,
• σ^+^ and σ^−^: represent the overestimation and the underestimation errors,
• Υ* and X_i_: are the value functions normalized in the interval [0,100].

The MUSA method provides a series of normalized indices for the in-depth analysis of customers’ satisfaction measurement. These indices include the total satisfaction function, satisfaction indices as well as demanding indices [16,20].

**Fig. 6:**
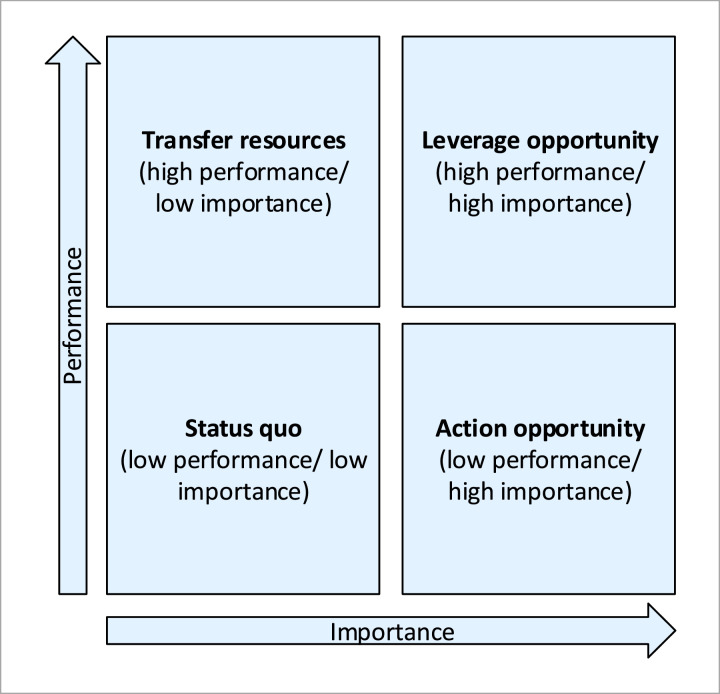
Action diagram elements [16,20].

Moreover, a series of action (performance – importance) diagrams are developed based on the MUSA method's results concerning the examined criteria and subcriteria level of satisfaction and demanding. These diagrams are called action diagrams and are divided into four quartiles based on the level of criteria and subcriteria performance (satisfaction) and importance (demanding) (Fig. 6).

Action diagrams’ quartiles are used in order to classify the actions an organization should take concerning the satisfaction criteria and subcriteria placed into them as follows [16,20]:

• Status quo area (low performance and low importance): generally, no action is required concerning the satisfaction criteria and subcriteria placed in this quartile.
• Leverage opportunity area (high performance/high importance): the satisfaction criteria and subcriteria placed in this quartile can be an organization's competitive advantage.
• Transfer resources area (high performance/low importance): organization's resources may be better used elsewhere than in the improvement of the satisfaction criteria and subcriteria placed in this quartile.
• Action opportunity area (low performance/high importance): attention should be paid into the improvement of these satisfaction criteria and subcriteria.

Thus, action diagrams are developed through the combinations of satisfaction criteria and subcriteria performance and importance levels and they are similar with a SWOT analysis as they may represent the strong and the weak points of an organization, indicating the priorities in customers’ satisfaction dimensions improvements [21].

## Ethics Statement

The authors informed all the potential participants that they will have to answer a total of 4 questionnaires, 1 at the end of each semester of the survey. Furthermore, the potential participants were informed that the survey was voluntary and completely anonymous.

## CRediT Author Statement

**Dimitrios Drosos:** Conceptualization, Methodology, Resources; **Michalis Skordoulis:** Investigation, Methodology, Supervision; **Nikos Tsotsolas:** Software, Methodology, Supervision; **Grigorios Kyriakopoulos:** Data curation, Methodology, Writing - review and editing; **Eleni Gkika:** Visualization, Resources, Writing - review and editing; **Faidon Komisopoulos:** Visualization, Resources, Writing - review and editing.

## Declaration of Competing Interest

The authors declare that they have no known competing financial interests or personal relationships which have or could be perceived to have influenced the work reported in this article.
